# Magnetic resonance imaging of intracranial anomalies in pregnancies complicated by twin anemia-polycythemia sequence

**DOI:** 10.1007/s00234-024-03373-4

**Published:** 2024-05-08

**Authors:** Stefano Tricca, Cecilia Parazzini, Chiara Doneda, Filippo Arrigoni, Mario Tortora, Mariano Lanna, Daniela Casati, Stefano Faiola, Andrea Righini, Giana Izzo

**Affiliations:** 1 Department of Pediatric Radiology and Neuroradiology, V. Buzzi Children’s Hospital, Via Castelvetro 32, 20154 Milan, Italy; 2 Department of Diagnosis and Treatment Services, Azienda Ospedaliero Universitaria Maggiore Della Carità, RadiodiagnosticsNovara, Italy; 3 Department of Advanced Biomedical Sciences, Neuroradiology Unit, University “Federico II”, Naples, Italy; 4 Fetal Therapy Unit “U. Nicolini”, V. Buzzi Children’s Hospital, University of Milan, Milan, Italy

**Keywords:** Fetal MRI, TAPS, Monochorionic twins, Laser surgery

## Abstract

**Purpose:**

To describe fetal brain Magnetic Resonance Imaging (MRI) findings in a large series of monochorionic (MC) pregnancies complicated by Twin Anemia-Polycythemia Sequence (TAPS) prenatally diagnosed, so to characterize the potential intracranial complications associated with this condition, their frequency and potential treatment options.

**Methods:**

This is a retrospective study of MC twin pregnancies complicated by TAPS and undergone fetal MRI in a single institution from 2006 to 2023.

MRI control was performed and post-natal ultrasound (US) or MRI were available.

**Results:**

1250 MC pregnancies were evaluated in our institution. 50 pregnancies (4%) were diagnosed with TAPS, 29 underwent a fetal brain MRI.

13/29 pregnancies (44.8%) demonstrated brain findings at MRI in at least a twin. Neuroradiological findings were detected in 14/57 twins (24.6%).

We detected four main categories of findings: hemorrhagic lesions, T2-weighted white-matter hyperintensities (WMH), brain edema-swelling and venous congestion. Nineteen findings were present in the anemic and three in the polycythemic twins, with a statistically significant ratio between the two groups (p-value = 0.01).

Intrauterine MRI follow-up demonstrated the sequalae of hemorrhagic lesions. A complete regression of brain swelling, veins prominence and T2-WMHs was demonstrated after treatment. Postnatal imaging confirmed prenatal features.

**Conclusions:**

Our work demonstrates that TAPS-related MRI anomalies consisted in edematous/hemorrhagic lesions that occur mostly in anemic rather than in polycythemic twins. Fetoscopic laser surgery could have a potential decongestant role.

Therefore, prenatal MRI may help in counselling and management in TAPS pregnancies, especially for the planning of therapy and the monitoring of its efficacy.

**Supplementary Information:**

The online version contains supplementary material available at 10.1007/s00234-024-03373-4.

## Introduction

Twin anemia–polycythemia sequence (TAPS) is a specific complication of mono-chorionic (MC) twins occurring spontaneously in 3–5% of cases or after an incomplete coagulation of anastomosis with laser surgery for treatment of twin-to-twin transfusion syndrome (TTTS), with a rate up to 16%. The most validated hypothesis on its pathogenesis is the presence of small (< 1 mm diameter) artero-venous anastomosis through which blood flows without large amount of volume as it occurs in TTTS cases, so to give rise to anemia-polycythemia without oligo-polyhydramnios sequence (TOPS) [1–3].

Prenatal diagnosis of TAPS is defined by an increased middle cerebral artery peak systolic velocity (MCA-PSV) in the donor twin and a lower one in the recipient without signs of TOPS, with a progression of severity defined by a recently proposed staging [4, 5]. Based on the new classification, TAPS pre-natal diagnosis is defined as a difference of delta MCA‐PSV > 0.5 MoM; without signs of fetal compromise, while cardiovascular compromise of the donor with abnormal flow in the umbilical artery or ductus venosus occurs in stage 3, hydrops in stage 4 and intrauterine death (IUD) of one or both fetuses in stage 5 [6].

TAPS treatments include induced preterm delivery; intrauterine blood transfusion (IUT) with or without partial exchange transfusion (PET); fetoscopic laser coagulation of placental anastomoses; selective feticide; or expectant management. Nevertheless, the most suitable treatment for this condition is still controversial, and perinatal mortality is still high in advanced stages, as a large international cohort study has recently confirmed [7].

Perinatal morbidity rate in TAPS is not well known, reflecting the heterogeneous nature of this disease [8, 9]. Limited knowledge on optimal management and prognosis of this condition restricts adequate parental counseling and informed decision making. Moreover, literature regarding prenatal brain imaging in pregnancies complicated by TAPS is scant [3].

The aim of this study is to describe fetal brain magnetic resonance imaging (MRI) anomalies resulting from the revision of a relatively large series of MC pregnancies complicated by pre-natal TAPS, so to characterize in detail the intracranial complications associated with this condition in the anemic and polycythemic group pre- and post-treatment.

## Materials and methods

### Study design and sample recruitment

This is a retrospective study of MC twin pregnancies complicated by pre-natal TAPS that underwent fetal MRI in a single institution between 2006 and 2023.

The candidates were recruited as clinical cases with ethical approval for retrospective review of clinical notes and MRI by the Milano Area B Ethics Committee (n. 2292/2016 protocol approval code). Informed consent to the research and to publication of the results was obtained.

The diagnosis of TAPS was defined by prenatal ultrasound (US) criteria during surveillance of MC twins through bi-weekly check-up, or after weekly longitudinal monitoring following laser surgery for TTTS. Pre-natal TAPS diagnosis was defined as a difference of delta MCA‐PSV > 0.5 MoM between the two fetuses according to the new classification system [6].

After TAPS diagnosis, a fetal MRI was performed within two weeks from US in cases with suspected brain alterations and in high-grade TAPS. Moreover since 2016, the execution of fetal MRI after ultrasound diagnosis of TAPS has become part of our hospital protocol.

Our center is participating to a randomized trial for TAPS treatment and cases which did not accept a randomization were treated as follow: laser surgery between 16 and 26 weeks, as already described, IUT from 20 to 28 weeks when laser was considered not feasible, preterm delivery after 28 weeks or observation when parents would not allow any treatment. Selective foeticide was discussed beneath the legal term for termination of pregnancy in our country, which is less than 23-week gestation [10].

Follow-up fetal MRI scans were performed two weeks after treatment or in those fetuses with positive findings at previous examination. Post-natal imaging follow-up (cranial ultrasound or MRI) was available for most of the survivor twins.

Exclusion criteria included cases with very low images quality, known congenital or chromosomal anomalies or lost at peri-natal follow-up.

For each subject, we recorded demographic data, pregnancy characteristics, invasive procedure related data, pre- and post-natal brain findings detected by both fetal and post-natal MRI scans (Table 1).

**Table 1:** Demographic data of TAPS pregnancies

Characteristic	Cohort (n = 29)
TAPS type	
- Spontaneous	16 (55.2%)
- Post-Laser	13 (44.8%)
GA at MRI TAPS diagnosis	22 (20—27 w)
TAPS stage	
- I	5 (17.2%)
- II	17 (58.6%)
- III	6 (20.7%)
- IV	1 (3.4%)
TAPS treatment	
- Fetoscopic Laser	12
- Intrauterine Transfusion	2
- Selective Feticide	1
- Watch and wait	15
Mean GA at delivery	31,6 (Range 24—38 w)
IUD of one twin	16 (55.2%)
- Spontaneous	11
- Post-Laser TAPS	3
- Selective Feticide	2

TAPS: twin anemia-polycythemia sequence; GA: gestational age; MRI: magnetic resonance imaging; IUD: intrauterine death

### Fetal brain MR imaging

MR images were obtained using 3.0 Tesla scanner (Magnetom VIDA; Siemens Healthcare GmbH, Erlangen, Germany) or 1.5 Tesla system (Achieva; Philips Healthcare, Best, Netherlands). Fetal brain MRI protocol included Single-Shot Fast Spin Echo (ss-FSE) T2-weighted (3 mm thick, about 1 mm^2^ plane res.), steady-state free precession/true (FISP) sequences on three orthogonal planes, and axial T1-weighted FSE sequence. Only in some cases, axial single-shot Fluid Attenuated Inversion Recovery (ss-FLAIR) sequence, T2*-weighted gradient-echo (GRE) sequence and diffusion-weighted (DW) images (4 mm thick, b = 600) were also collected.

MRI exams were reviewed independently by two certified pediatric neuroradiologists with more than five-year experience in fetal neuroimaging to assess presence of parenchymal injury or any other intracranial abnormal finding. In case of disagreement, images were reviewed together and consensus was reached.

#### Post-natal brain MR imaging

Post-natal MRI exams were obtained using the same scanners of pre-natal imaging (3.0 T and 1.5 T) with a protocol that included 3D-T1 MPRAGE, axial and coronal T2- weighted FSE, DWI and susceptibility-weighted imaging sequence (SWI) sequence.

### Statistical analysis

Because of the relatively small sample size and the not-normal distribution of the data, measures of central tendency and dispersion were used to describe population’s characteristics.

All quantitative data, including brain parenchymal injuries and its evolution have been described with numbers and percentages.

To evaluate differences in brain findings among patients with different characteristics chi-squared test and Fisher exact test were used. A p-value less than 0.05 was considered statistically significant. All statistical analyses were performed in STATA 18.0 (StataCorp).

## Results

### Study Sample

**Fig. 1:**
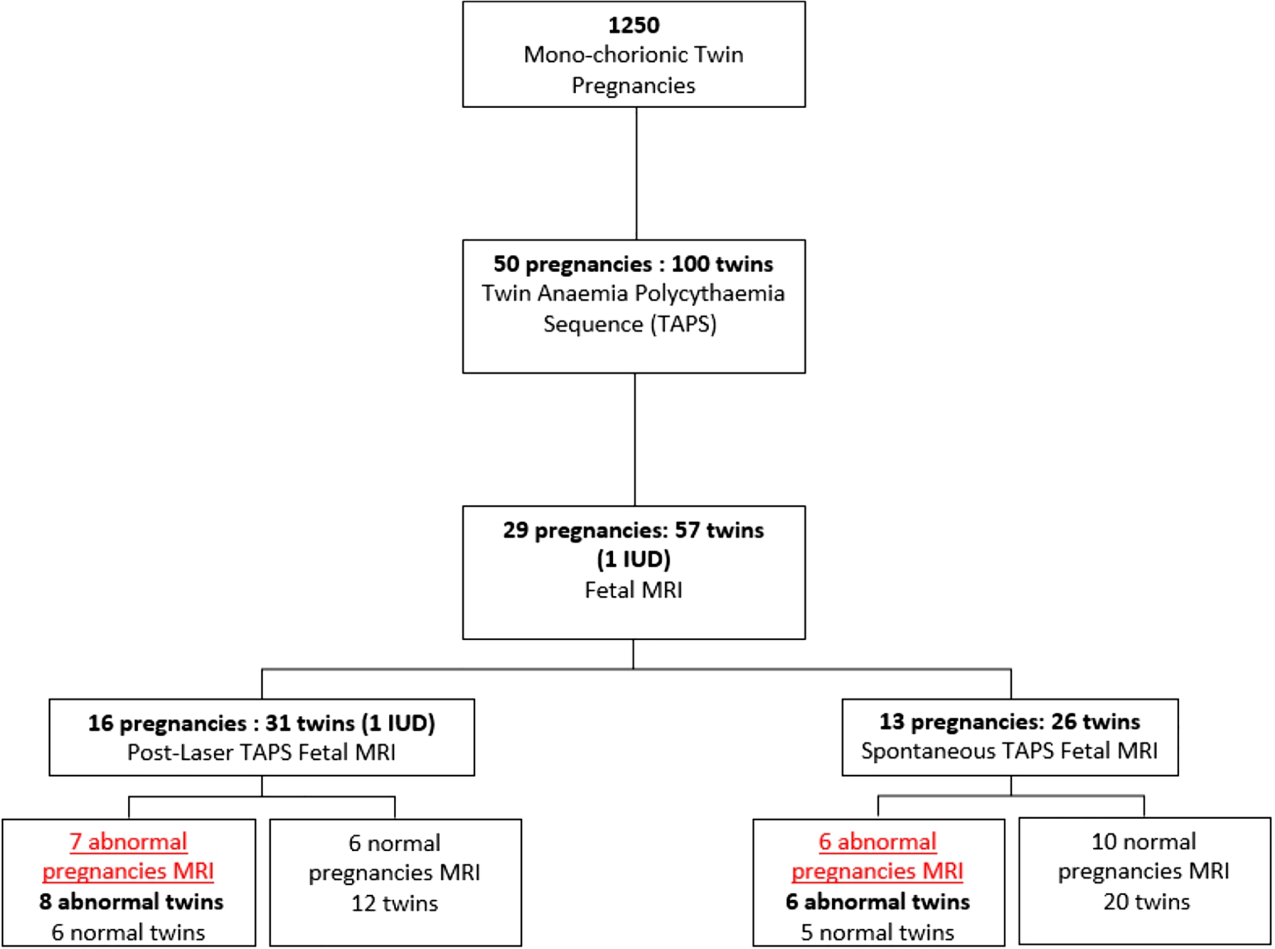
Case Selection IUD: intrauterine death; MRI: magnetic resonance imaging

Case selection is described in Fig. 1.

A total of 1250 MC-diamniotic pregnancies were evaluated in our Fetal Therapy Unit during the study period.

Overall, 50 pregnancies (100 fetuses, 4%) were diagnosed with TAPS. Of these, 29 underwent a fetal brain MRI for a total of 57 fetuses (55 at TAPS diagnosis, one fetus died, and a couple of twins only post treatment), and they represent the final sample. Table 1 summarizes the main characteristics of all pregnancies undergone MRI.

The mean gestational age (GA) at TAPS diagnosis was 22 weeks (sd 3.1; minimum 20 weeks; maximum 27 weeks). 16/29 (55%) were spontaneous TAPS and 13/29 (45%) were post-laser.

Fourteen (48.3%) pregnancies underwent treatment for TAPS, 11 were treated with fetoscopic laser procedure, one with intrauterine blood transfusion (IUT), one with both laser and IUT and only one with selective feticide. The remaining 15 were subjected to a wait and see protocol by serial US examinations due to high procedural risk or lack of maternal consent or due to low-grade TAPS and/or minimal brain lesion load that remained stable on ultrasound.

Thirteen MC pregnancies (44.8%) demonstrated abnormal intracranial findings at MRI in at least a twin; in particular, in 12 of these pregnancies a single fetus was affected, in one pregnancy both twins displayed abnormal findings, thus neuroradiological findings were detected in 14/57 twins (24.6%), 12 of these at TAPS diagnosis, and two at post-treatment control. In Table 2 are described case-by-case the characteristics of those pregnancies. At least one additional fetal MRI control was made with a mean interval of 3.5 weeks (sd 1.9 weeks; minimum one week; maximum seven weeks). Fetuses with normal intracranial findings at TAPS diagnosis were monitored by serial US; only three of them underwent a second MRI control after intrauterine death (IUD) of the co-twin.

**Table 2:** Subject characteristics and MR imaging examination findings

Pr	TAPS Type	TAPS Stage	US findings	GA at MRI	Anemic MRI Findings	Polycythemic MRI Findings	TAPS Treatment	Anemic Post Treatment MRI	Polycythemic Post Treatment MRI	GA birth Fetal Fate	Post- Natal Imaging (US/MR)
ID 01	S	III	A: IUGR	25	Brain swelling; venous prominence; peri ventricular T2w hyperintensity	Normal	Transfusion	Brain swelling; hematoma of the germinal matrix; periventricular radial T2w hypointensity	Normal	P: 34w A: IUD	P: Normal US
ID 02	S	III	A: IUGR, frontal in-homogeneity	30	/	/	Laser	Malacic cortical lesions	Normal	P: 34w A: selective feticide	P: Normal US
ID 03	L	II	A: IUGR	22	Brain swelling; peri ventricular T2w hyperintensity	Normal	Laser	Brain swelling regression	Normal	Both: 36w	P: Normal MR A: periventricular bilateral punctate lesions
ID 04	L	III	A: IUGR	27	IUD	Linear T2w hypointensity within the germinal matrix	Wait and see	/	/	P: 27w A: IUD	P(MR): ependymal hemosiderinic deposits; cortical sulci prominence; germinolitic cyst
ID 05	L	II	Normal	24	Brain swelling; hematoma of germinal matrix; periventricular radial T2w hypointensity	Normal	Laser	IUD	Normal	P: 29w A: IUD	P: Normal US
ID 06	S	IV	Normal	21	Normal	Normal	Laser	IUD	Venous prominence Ventricular asymmetry with stretched septum pellucidum	P: 24w A: IUD	P(MR): unilateral IVH
ID 07	L	II	A: periventricular hyperechoic area, IUGR	22	Brain swelling; hematoma of germinal matrix; periventricular radial T2w hypointensity; venous prominence; peri ventricular T2w hyperintensity	Normal	Laser	Brain swelling regression; ependymal hemosiderinic deposits; unilateral porencephalic sequelae and ex-vacuo VM	Normal	Both: 30w	P: Normal MR A: ependymal hemosiderinic deposits; unilateral porencephalic sequelae
ID 08	S	I	Normal	22	Linear T2 hypointensity within the germinal matrix	Normal	Wait and see	Ependymal hemosiderinic deposits	Normal	Both: 35w	P(MR): hemorrhagic petechiae A(MR): IVH and hemorrhagic petechiae
ID 09	S	II	A: IUGR	22	Venous prominence	Venous veins prominence	Laser	Normal	Normal	Both: 34w	Both Normal MR
ID 10	S	II	Normal	21	Brain swelling; Venous prominence	Normal	Laser	Brain swelling regression	Normal	Both: 24w	Both Normal MR
ID 11	L	II	Both IUGR; P: unilateral VM	20	Normal	Linear T2w hypointensity within the germinal matrix; mild unilateral VM	Wait and see	Normal	Ependymal hemosiderinic deposits; mild unilateral VM	Both: 35w	A: Normal MRI P: minimal ependymal hemosiderinic deposits and unilateral parenchymal sequelae
ID 12	L	II	A: brain swelling	25	Brain swelling; peri ventricular T2w hyperintensity; venous prominence	Normal	Wait and see	IUD	Normal	P: 34w A: IUD	P: Normal US
ID 13	S	I	A: IUGR	21	Venous prominence	Normal	Wait and see	Venous prominence	Normal	Both at 33	Both Normal MRI

A: anemic; GA: gestational age; ID: identification code of pregnancy; IUD: intra-uterine death; IUGR: intra-uterine growth restriction; IVH: intra-ventricular hemorrhage; L: post-laser; MR: magnetic resonance; P: polycythemic; Pr: pregnancy; S: spontaneous; TAPS: twin anemia-polycythemia sequence; US: ultrasound; WM: white matter; VM: ventriculomegaly

Overall,15 IUDs occurred (25.9%) and the surviving twins (74.1%) were born at a mean GA of 31.6 weeks (SD 4.1; minimum 24 weeks; maximum 38 weeks).

All fetuses of our sample underwent post-natal control. In almost all of those with intracranial findings at fetal MRI, a post-natal MRI was preferably performed within one month from delivery. Fetuses without TAPS related brain complications during fetal life underwent US follow-up.

**Fig. 2:**
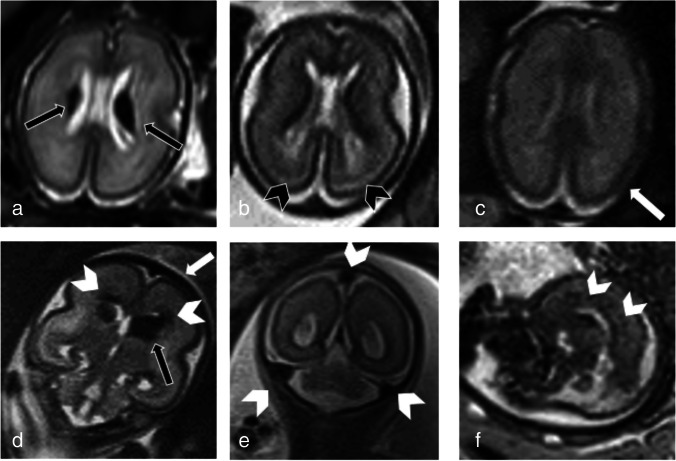
Twin Anemia-Polycythemia Sequence (TAPS) intracranial Magnetic Resonance (MRI) main findings in six different exemplificative cases: a) axial ss-FSE T2-weighted image from 24 week study showing bilateral intraventricular haemorrhage—IVH (black arrows), b) axial ss-FSE T2-weighted section from 22 week study showing bilateral para-trigonal blurred areas of higher T2-weighted signal (black arrow-heads), c) axial ss-FSE T2-weighted section from 25 week study showing cortical liquoral space obliteration due to brain swelling (arrow), d) coronal ss-FSE T2-weighted sequence from 22 week study showing germinal matrix hematoma (black arrow) with periventricular radial T2-weighted hypointensities (white arrow-head), brain swelling (white arrows), e–f) coronal and sagittal ss-FSE T2-weighted sections from 22 and 21 week study showing prominence of transverse and sagittal sinuses (arrow-heads in case in e), and deep medullary vein system (arrow-heads in case in f)

TAPS type (spontaneous or post-laser) was not significantly different between patients with or without prenatal MRI anomalies with a Chi-square test of 0.775 (*p*-value 0.38; Fisher's exact test 0.47). Similarly TAPS stage also resulted not different with a t-test of 0.849. (p-value 0.20) Fig. 2.

### MRI findings

Following TAPS diagnosis through US, among 12/55 fetuses with abnormal intracranial MRI findings (21.8%), nine were anemic and three polycythemic. Most of the twins showed more than one anomaly.

Brain MRI findings are summarized on Table 2.

The main MRI findings were:

- Hemorrhagic lesions, with different characteristics, in five subjects (8.8%). In particular, we detected three (ID04, ID08 and ID11) unilateral linear T2-weighted hypointensities along the ventricle margin (low grade germinal hemorrhage), two (ID05, ID07) bilateral hematomas within the germinal matrix (higher grade hemorrhage), and two (ID05, ID07) periventricular radial T2-weighted hypointensities (as small hemorrhages in the deep medullary veins territory), one was unilateral and one bilateral. Altogether, these findings were present in three anemic and in two polycythemic fetuses.
- T2-weighted periventricular white matter hyperintense areas (WMHs) in four patients (ID01, ID03, ID07 and ID12) (7%). This finding is suggestive of periventricular areas of parenchymal vasogenic-interstitial edema. This pattern was recognized only in the anemic fetuses.
- Diffuse generalized brain swelling in six patients (ID01, ID03, ID05, ID07, ID10 and ID12) (10.5%). This entity consists of parenchymal enlargement with consequent obliteration of the periencephalic CSF spaces in fetuses with normal skull biometry. Also, this finding was detected only in anemic fetuses.
- Prominence of dural sinuses and deep medullary veins in 7 patients (ID01, ID07, ID09, ID10, ID12 and ID13) (12.3%). Venous prominence was recognizable as unequivocable marked dilation of the dural sinuses and/or prominence of the deep medullary veins, recognizable as periventricular radially oriented venous structures. Venous findings were detected in 6 anemic fetuses and only in one polycythemic.
- Unilateral ventriculomegaly: unilateral, found in one polycythemic patient (ID11) (1.8%).

All findings are summarized in Table 3 divided between anemic and polycythemic twins. Given that most of the twins showed more than one type of anomaly, a total of 19 intracranial findings were present in the anemic twins and only three in the polycythemic ones, with a **statistically** significant ratio between the two groups (chi-square of 7.42; p-value = 0.01 and Fisher exact test of 0.01).

**Table 3:** Fetal MRI findings in anemic and polycythemic groups

Fetal MRI findings	Anemic Twins	Polycythemic Twins	p values
Hemorrhagic lesions	3 (60%)	2 (40%)	0.38
T2-WM hyperintense areas	4 (100%)	0 (0%)	0.02
Brain swelling	6 (100%)	0 (0%)	0.01
Venous prominence	6 (85,7%)	1 (14,3%)	0.02
TOTAL	**19 (86.4%)**	**3 (13.6%)**	**0.01**

Detailing the different type of findings according to anemic and polycythemic groups, a statistically significant difference was also observed about cerebral swelling (chi-square of 8.86; p-value = 0.01 and Fisher exact test of 0), white matter T2-weighted hyperintense (chi-square of 5.28; p-value = 0.02 and Fisher exact test of 0.04) and venous congestion (chi-square of 5.79; p-value = 0.02 and Fisher exact test of 0.03).

The difference in hemorrhagic lesions (chi-square of 0.38; p-value = 0.54 and Fisher exact test of 0.64) did not result statistically significant.

### Intrauterine MRI follow-up

Intrauterine MRI longitudinal findings of our sample are also summarized case-by-case in Table 2.

In particular, all three cases (ID04, ID08 and ID11) with linear ventricular margin hemorrhages evolved into intraventricular hemosiderine depositions associated with minimal unilateral ventricular dilatation in one patient (ID11). In the survivor fetus (ID07) with germinal matrix hemorrhage associated with periventricular medullary veins hemorrhages, after laser therapy, an evolution in a focal area of ​​periventricular malacia was found. An anemic twin (ID01) with bilateral prominence of deep medullary veins, after transfusion developed bilateral hemorrhagic lesions with blood along the germinal matrix associated with periventricular radial T2-weighted hypointensities.

**Fig. 3:**
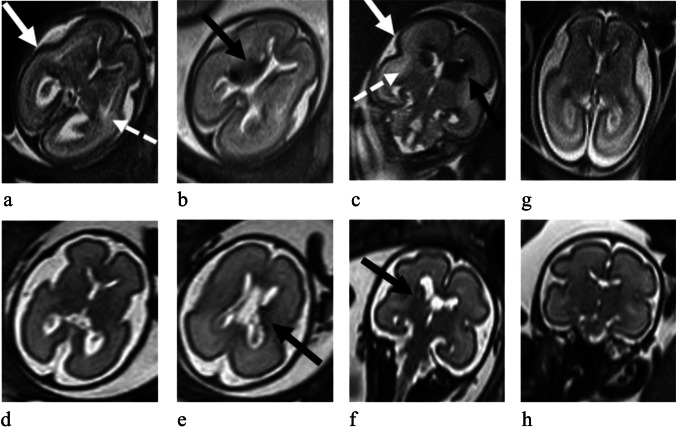
Axial (a and b) and coronal (c) ss-FSE T2-weighted sections from 22 week study of case ID07 showing in the anemic twin cortical cerebrospinal fluid (CSF) space reduction due to transient brain swelling (white arrows) and areas of high T2-weighted signal due to parenchymal edema (dotted arrows); black arrows point at germinal matrix hematoma associated with periventricular radial T2W hypointensities. g) unremarkable polycythemic co-twin for comparison. Axial (d and e) and coronal (f) from 28 week follow-up study of the anemic twin after laser treatment, depicting regression of brain swelling and of parenchymal edema areas, while periventricular sequelae of intraventricular haemorrhage (IVH) are present (black arrows). h) polycythemic co-twin follow-up is also depicted

In all patients who survived after laser treatment, we observed complete regression of the brain swelling, sinuses and medullary veins prominence and T2-weighted periventricular WMHs, as shown in Fig. 3.

In the only anemic patient (ID01) treated with transfusion, brain swelling persisted.

In only anemic patient (ID13) without treatment sinuses and deep medullary veins congestion persisted.

On the contrary, a survivor polycythemic twin (ID06) developed venous sinuses and deep medullary veins prominence in the MRI control after laser therapy and IUD of the anemic twin.

Finally, in the pregnancy (ID02) undergone MRI control only after laser therapy (performed one month before), the anemic twin showed bilateral parasagittal malacic lesions.

### Post-natal follow-up

All live-born twins had follow-up imaging in their first month of life (Table 2).

In particular, 16/20 twins underwent a post-natal MRI scan, while the remaining four underwent serial ultrasound exams as reported in Table 2.

In most cases, postnatal imaging confirmed the prenatal features and showed the expected evolution of prenatal findings, such as ependymal deposits of hemosiderine or germinolytic cyst (ID04) in low grade germinal hemorrhage.

Conversely ID03 (anemic twin) showed on MRI multiple punctate periventricular lesions consistent with periventricular leukomalacia (PVL), likely related to perinatal distress and/or preterm delivery.

ID06 (survivor polycythemic twin) showed a more recent intraventricular hemorrhage also related to perinatal distress and/or preterm delivery, while the venous sinuses and deep medullary veins salience subsided at post-natal MRI.

ID08 twins developed multiple hemorrhagic parenchymal petechiae, which were likely to be referred to a superimposed alloimmune thrombocythemia.

## Discussion

We reported the spectrum of TAPS related intracranial anomalies at different stages, from onset, after therapy, till postnatal follow-up, and we showed that periventricular hemorrhages, parenchymal focal periventricular edema, generalized brain edema-swelling, and venous congestion are the main intracranial complications. Although evidence of such intracranial anomalies in this condition have been reported in few case reports or as small series (10–12), our larger cohort demonstrates how anemic twins are much more susceptible to intracranial injuries, and, more importantly, how some of those brain edema-swelling and venous congestion features can be reversible after treatment. We detected MRI anomalies in at least a twin in 13/29 MC pregnancies, for a total of 14/57 twins (24.6%); twelve of these at time of diagnosis and two at post-treatment control.

The four main categories of MRI findings were: hemorrhagic lesions, areas of T2-weighted WM hyperintensity, brain edema-swelling, and venous congestion. Intracranial hemorrhages can be divided into intraventricular-germinal matrix subtle linear hemorrhagic lesions or large hematomas. In the three cases (ID04, ID08 and ID11) in which only a low-grade intraventricular hemorrhage was present, the MRI control performed after any treatment, showed an evolution toward hemosiderine deposits. In the three fetuses with high grade intraventricular hemorrhage (ID05 and ID07 at TAPS diagnosis and ID01 at control after transfusion), periventricular and deep medullary vein territory hemorrhages coexisted. These findings evolved into a malacic cavity in one fetus (ID07) treated with laser therapy; the other two fetuses (ID01 and ID05) died, respectively after transfusion and laser treatment.

Parenchymal edema/swelling, areas of T2-weighted hyperintensity and venous congestion were often coexisting and associated with large intraventricular hemorrhages, compatible with general state of central venous hypertension hindering intracranial venous output. Venous dural sinuses prominence was also found as an isolated finding without generalized edema or hemorrhage (ID09, ID13), probably reflecting a less severe venous outflow impairment.

We found complete regression of brain swelling/edema and of the signs of intracranial venous congestion in almost all patients at MRI follow-up after laser treatment. This leads to suppose a potential decongestant role of fetoscopic laser treatment, whose intracranial positive effect has not been highlighted so far. On the contrary, in one anemic fetus (ID13), who did not undergo treatment, venous salience persisted, as well as in case ID01 where the pre-existing edematous status of the anemic twin evolved in high grade hemorrhage after transfusion. Only in one polycythemic patient (ID06), venous congestion developed after laser and IUD of the co-twin.

Interestingly, in our sample, these findings were found with a significantly higher frequency in anemic twins than in polycythemic ones. This association between fetal anemia and brain lesions might be related to a combination of different factors present in TAPS: blood decreased oxygen-carrying capacity and lower anemic related viscosity in association to increased blood flow; that may result in heart failure (hyperkinetic heart overload) and increased venous central pressure with reduction of venous return. Moreover, the resulting hydrops can lead to hypovolemic hypotension and hyperdynamic circulation, as well as hypoxia/ischemia can contribute to damage cerebral vessels and brain development in donor twins; the alteration of coagulation may also play a role in this pathological circle [11].

Studies regarding the neurological complications of severe fetal anemia and circulation overload due to different etiologies have reported either hemorrhagic or ischemic lesions resulting from MR or US imaging [11–13]. The intracranial features we observed in our anemic twins are corroborated also by the observations reported by Doneda et al. in a cohort of fetal MRI cases depicting intra and extra-parenchymal venous engorgement in fetuses severely anemic and with consequent hyperkinetic heart overload [14]. The predominant pathogenetic hypothesis regarding hemorrhagic damage in polycythemic fetuses is that vascular hyperviscosity can lead to the consumption of coagulation factors, potentially determining the development of cerebral sinovenous thrombosis [8].

Also, the well-known relative immaturity of deep venous intraparenchymal system of the early fetal age has to be taken into account when considering the risk of hemorrhages associated with central venous system congestion in both anemic and polycythemic twins.

In our sample, only one fetus (ID02) showed a probable arterial ischemia with a pattern consisting of bilateral cortical-subcortical parasagittal malacic lesions; however, this is the only pregnancy undergone MRI only after laser treatment (one month later) and therefore such lesions could be also related to the treatment itself [15].

In the only other work reporting a prenatal MRI series of TAPS cases [9], 7/46 fetuses were affected by ischemic or hemorrhagic lesions, with a higher prevalence of cerebral injury in polycythemics (71.4%). Other two case-reports described scant hemorrhagic lesions in the recipient twins, the most recent in a post-natal MRI after the development of a disseminated intravascular coagulation [16, 17].

Instead, our data are consistent with some larger and recent studies. Results of an international TAPS registry have shown that in spontaneous TAPS, perinatal mortality occurs in 12% of donors (vs 5% of recipients), suggesting anemic status as an independent risk factor for spontaneous perinatal mortality [18].

A recent article also reported anemia as an independent risk factor for neurological developmental impairment (NDI) and a rate of four times greater risk of NDI in donor compared to recipient co-twins. The same study also reports an increased risk of cognitive delay and deafness in anemic twins [19].

Another large international study reported higher perinatal mortality and neonatal morbidity in anemic donors than in recipient twins (18% in donors compared to 3% of recipients) [20]. However, these works did not report the neuro-imaging alterations affecting the patients.

We performed MRI soon after TAPS diagnosis in most cases; however, in one case MRI was achieved after IUD of the anemic twin (ID04), potentially representing a risk factor for brain lesions in the co-twin as Conte et al [21] demonstrated in an observational study where they considered brain lesions to be mostly of ischemic nature, while in our case we observed a low-grade hemorrhagic lesion.

The main limitations of our study are represented by its retrospective nature; by the heterogeneity of timing of diagnostic/therapeutic setting, although the clinical management variability in timing and decision making is intrinsic in this disease; by the relatively small sample size, although the largest so far reported by mean of prenatal MRI investigation. Another limitation could be that fetal MRI studies were performed at scanners with different magnetic fields (1.5 vs 3 T) and this may have had some interference with lesion detection, at least about some minor ones. Similarly, the lack of DWI and GRE images in the majority of studies may have limited the sensitivity to smaller hemorrhagic or very acute ischemic lesions.

Due to the relatively small number of cases, we could not perform a sub-group statistics dividing cases imaged before and after 2016, when we shifted towards a more extensive MRI application on MC twins. This could have biased our results towards a higher prevalence of TAPS-related intracranial findings.

A standardized diagnostic protocol including serial MRI exams in a larger cohort of MC pregnancies with TAPS diagnosis is suggested to confirm our results. It would be also suitable to include in such protocol the diffusion weighted sequences to better highlight acute lesions, and which were not included consistently in our retrospective study. Finally, it is desirable future research focusing on the two- or five-year outcome of the cases with imaging anomalies, including neurological evaluation and standardized scales.

In conclusion, our work showed in detail the edematous/hemorrhagic lesions in a relatively large cohort of MC twins pregnancies complicated by TAPS and how they occur more frequently in anemic than in polycythemic twins. Moreover, post-treatment MRI allowed to demonstrate the reversibility of some of these findings potentially related to the positive effect of fetoscopic laser-application.

Prenatal MRI may help in the management of TAPS pregnancies, especially for the planning of therapy and the monitoring of its efficacy.

### Supplementary Information

Below is the link to the electronic supplementary material.

Supplementary file1 (DOCX 264 KB)
