# Supplementary material for: Magnetic resonance imaging of intracranial anomalies in pregnancies complicated by twin anemia-polycythemia sequence
Source: Neuroradiology. 2024 May 8;66(7):1213–23. doi: 10.1007/s00234-024-03373-4 (PMC11150324; doi:10.1007/s00234-024-03373-4)
Supplement: Supplementary file 1 — Supplementary file1 (DOCX 264 KB) [file 234_2024_3373_MOESM1_ESM.docx]

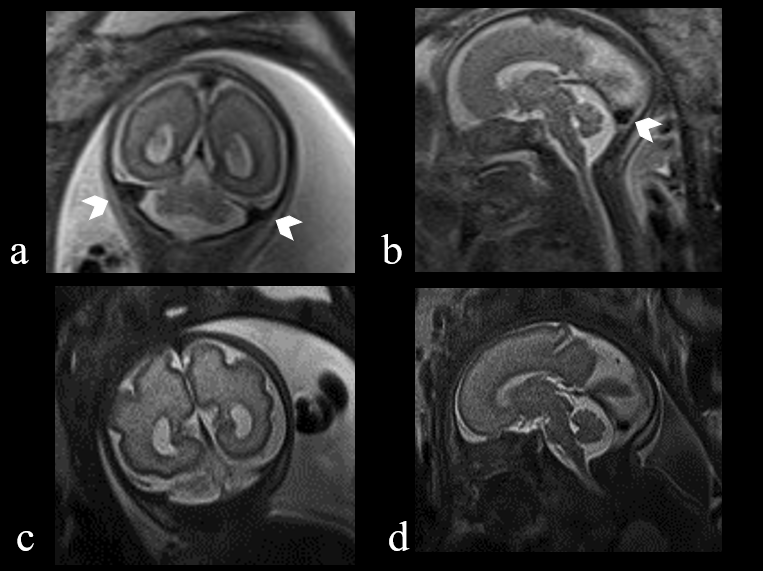


Figure 4

a and b) Coronal and sagittal ss-FSE T2-weighted sections from 22 week study in the anemic twin (ID 09 of Table 2) showing prominence of transverse sinuses and torcular (arrow-heads); c and d) corresponding sections from 28 week follow-up study showing regression of venous sinuses dilation.
